# Supplementary material for: A fault-intrusion-tolerant system and deadline-aware algorithm for scheduling scientific workflow in the cloud
Source: PeerJ Comput Sci. 2021 Nov 2;7:e747. doi: 10.7717/peerj-cs.747 (PMC8576554; doi:10.7717/peerj-cs.747)
Supplement: Supplemental Information 6 [file peerj-cs-07-747-s006.zip › Myproject1.1/docs/org/cloudbus/cloudsim/power/package-summary.html]

org.cloudbus.cloudsim.power


JavaScript is disabled on your browser.


- Overview
- Package
- Class
- Use
- Tree
- Deprecated
- Index
- Help

- Prev Package
- Next Package

- Frames
- No Frames

- All Classes

# Package org.cloudbus.cloudsim.power

- Class Summary

  | Class | Description |
  | --- | --- |
  |  |  |
  | --- | --- |
  | PowerDatacenter | PowerDatacenter is a class that enables simulation of power-aware data centers. |
  | PowerDatacenterBroker | A broker for the power package. |
  | PowerDatacenterNonPowerAware | PowerDatacenterNonPowerAware is a class that represents a non-power aware data center in the context of power-aware simulations. |
  | PowerHost | PowerHost class enables simulation of power-aware hosts. |
  | PowerHostUtilizationHistory | The class of a host that stores its CPU utilization history. |
  | PowerVm | The class of a VM that stores its CPU utilization history. |
  | PowerVmAllocationPolicyAbstract | The class of an abstract power-aware VM allocation policy. |
  | PowerVmAllocationPolicyMigrationAbstract | The class of an abstract power-aware VM allocation policy that dynamically optimizes the VM allocation using migration. |
  | PowerVmAllocationPolicyMigrationInterQuartileRange | The Inter Quartile Range (IQR) VM allocation policy. |
  | PowerVmAllocationPolicyMigrationLocalRegression | The Local Regression (LR) VM allocation policy. |
  | PowerVmAllocationPolicyMigrationLocalRegressionRobust | The Local Regression Robust (LRR) VM allocation policy. |
  | PowerVmAllocationPolicyMigrationMedianAbsoluteDeviation | The Median Absolute Deviation (MAD) VM allocation policy. |
  | PowerVmAllocationPolicyMigrationStaticThreshold | The Static Threshold (THR) VM allocation policy. |
  | PowerVmAllocationPolicySimple | This a simple class representing a simple VM allocation policy that does not perform any optimization of the VM allocation. |
  | PowerVmSelectionPolicy | The class of an abstract VM selection policy. |
  | PowerVmSelectionPolicyMaximumCorrelation | The Maximum Correlation (MC) VM selection policy. |
  | PowerVmSelectionPolicyMinimumMigrationTime | The Minimum Migration Time (MMT) VM selection policy. |
  | PowerVmSelectionPolicyMinimumUtilization | The Minimum Utilization (MU) VM selection policy. |
  | PowerVmSelectionPolicyRandomSelection | The Random Selection (RS) VM selection policy. |

- Overview
- Package
- Class
- Use
- Tree
- Deprecated
- Index
- Help

- Prev Package
- Next Package

- Frames
- No Frames

- All Classes
